# Spatial-temporal simulation for hospital infection spread and outbreaks of *Clostridioides difficile*

**DOI:** 10.1038/s41598-023-47296-1

**Published:** 2023-11-16

**Authors:** Denisse Kim, Bernardo Canovas-Segura, Amaya Jimeno-Almazán, Manuel Campos, Jose M. Juarez

**Affiliations:** 1 Med AI Lab, University of Murcia, Campus Espinardo, 30100 Murcia, Spain; 2 Murcian Bio-Health Institute (IMIB-Arrixaca), El Palmar, 30120 Murcia, Spain; 3 Internal Medicine Service, Infectious Diseases Section, Hospital Universitario Santa Lucía, Cartagena, Spain

**Keywords:** Computer science, Software, Bacterial infection, Clostridium difficile

## Abstract

Validated and curated datasets are essential for studying the spread and control of infectious diseases in hospital settings, requiring clinical information on patients’ evolution and their location. The literature shows that approaches based on Artificial Intelligence (AI) in the development of clinical-support systems have benefits that are increasingly recognized. However, there is a lack of available high-volume data, necessary for trusting such AI models. One effective method in this situation involves the simulation of realistic data. Existing simulators primarily focus on implementing compartmental epidemiological models and contact networks to validate epidemiological hypotheses. Nevertheless, other practical aspects such as the hospital building distribution, shifts or safety policies on infections has received minimal attention. In this paper, we propose a novel approach for a simulator of nosocomial infection spread, combining agent-based patient description, spatial-temporal constraints of the hospital settings, and microorganism behavior driven by epidemiological models. The predictive validity of the model was analyzed considering micro and macro-face validation, parameter calibration based on literature review, model alignment, and sensitive analysis with an expert. This simulation model is useful in monitoring infections and in the decision-making process in a hospital, by helping to detect spatial-temporal patterns and predict statistical data about the disease.

## Introduction

In recent years, Artificial Intelligence (AI) techniques have demonstrated their potential to implement effective data-driven clinical decision support systems^1,2^. These techniques are strongly dependent on the volume and quality of available clinical data. However, its development is limited due to the problems associated with the use of sensitive and sometimes incomplete clinical data and, for example in the case of machine and deep learning, the low interpretability of the models generated (“black box problem”)^3,4^. The growing concern of the medical AI community by the observed lack of reproducibility and interpretation of these models have entailed the study of more trustworthy and ethical guidelines for AI-based systems^5,6^.

Some approaches have been proposed to address this issue. Among them, we find the anonymization of real health data and the development of digital twins - dynamic virtual representations of physical objects -. However, both of these techniques share many of the same issues and challenges faced by AI and data analytics^7,8^, such as availability of quality data, risk of bias, the privacy of individuals (e.g., through data triangulation techniques^9^), ethical behavior in the collection and use of data, confidentiality and consent, among others^7^.

Realistic data simulations can be part of the solution to this problem. Although it is not possible to simulate all the real-world factors that can influence a parameter of study or a specific situation (e.g. changes in decision making, errors, etc.), they have a twofold benefit: from a public health perspective, they enable predictive analysis and an early evaluation of hospital policies; from a medical AI perspective, simulated datasets are helpful for building and evaluating future AI techniques in a more fair and trustworthy way^10^.

The constant increase in the prevalence of healthcare-associated infections (HAIs) caused by multi-resistant microorganisms is currently posing a challenge and is one of the main concerns in public health. Bacteria and other pathogens are capable of evolving and becoming resistant to the drugs that are used to fight them, turning into what is known as multidrug-resistant microorganisms (MDR-microorganism)^11^. This resistance complicates the treatment and increases its severity, mortality, and risk of spread^12^. Therefore, a priority issue is to control and prevent MDR bacterial infections, since they involve rises in healthcare costs and a threat to society. Healthcare systems must have the necessary means to be able to evaluate the presence of these infections within hospitals. To this end, the spatial structure of a hospital and the physical distribution of patients over time play important roles in detecting outbreaks and preventing their spread.

The increasing need to study MDR-bacterial infections has led to a variety of computational model implementations of different types. The ones that stand out in the literature are network-based, agent-based, and compartmental models.

**Network-based models** are developed to study the movement and contact between patients, and therefore, the transmission of diseases, but space does not play any role other than informative^13–15^. From an epidemiological perspective, **compartmental models** are classic frameworks for quantifying disease transmission and studying the application of intervention strategies^16–18^. The population is divided into labeled compartments, they can progress from one to another and, depending on the labels, there are different approaches (e.g. SIR, SIS, SEIR, etc.). However, these approaches do not represent explicitly individual contact within a population but rather show dynamics on a large scale^19^.

In contrast to these, **agent-based models** are used to study the dynamic processes that involve agents’ interactions with each other and with the environment^20^. In such dynamic processes, both individuals and environments can change and adapt over time, which makes these models suitable for discovering spatial patterns derived from the results of interactions at individual level^20^. They can also be applied to the study of dynamic processes related to the effects of space on health or to the specific processes that are believed to lead to the observed empirical regularities. In a study conducted by Willem et al.^21^, they identified 698 papers about agent-based implementations with infectious diseases, of which 89 worked with bacterial infections for different purposes. Another systematic review^22^ studied 372 papers on different simulation approaches applied in COVID-19 research and found out that agent-based models were the most used and covered more research areas than the others.

Several studies have used agent-based approaches to analyze the transmissions of infectious diseases in hospitals and the effects of control strategies. Codella et al.^23^ developed an agent-based model combined with a Markov model to study the transmission of *Clostridioides difficile* (CD) and analyzed the performance of several control measures in a mid-size hospital. Nelson et al.^24^ developed an economic analysis of the strategies applied in a hospital to control the transmission of CD and conducted probabilistic sensitivity analyses in which all parameter values were allowed to vary simultaneously through 2nd order Monte Carlo simulations. Lee et al.^25^ presented a software tool that generates an agent-based simulation to study the spread and control of infectious diseases in any healthcare ecosystem, and they evaluated the performance using real datasets. Haber et al.^26^ developed a simulation to explore different regimes that use second-line antibiotics - those given when the initial treatment is not effective or is no longer effective - to successfully treat and reduce the resistance frequency to other drugs. They evaluated this model with several runs to predict the effectiveness of various treatment strategies.

In this paper we present a simulation model with the aim of obtaining a reliable spatial-temporal dataset on the activity of hospitalized patients. This realistic simulator is a goal of a research project on eXplainable AI (see Acknowledgements) applied to the monitoring of infection spreads in hospitals caused by relevant bacteria. This intersection between spatial, temporal and epidemiological information is not easy to achieve by other means and is, in turn, necessary for studies in this field. The contribution of this work is a simulation model that combines (1) a compartmental model to represent the evolution of bacterial infections (macro-model); (2) an agent-based model for the dynamics and spread of the infection as well as the individual actions (micro-model); and (3) spatial-temporal constraints defined by the hospital infrastructure, through the representation of its layout, cleaning policies, and staff shifts.

## Methods

Our proposal is funded on basic principles of computer simulation, and also on epidemiological modelling. We combine an agent-based model with an epidemiological compartmental model and the policies and structure of a healthcare environment to study the dynamics of a population within the hospital and analyze the spread and outbreaks of an infectious disease in that population. In this section, we describe the simulation model characteristics following the 7 steps of the Overview, Design concepts and Details (ODD) protocol^27^:

### Observed phenomenon and simulation assumptions

The phenomenon to simulate is a bacterial infection spread in a hospital setting. Due to the complexity because of the number of unknown interactions and the lack of a complete picture, there is no comprehensive ground of truth in the literature at the moment of submission.

This proposal is based on a discrete event time approach. The mathematical relations of the phenomenon to simulate are unknown and cannot be solved analytically. As far as we are concerned, there is no knowledge about continuous time description in the literature, thus differential equation models are not possible. For this research, we have made the following assumptions, which were validated by clinical experts and supported by recent medical literature:

- The simulation model concerns the dynamics of a single microorganism.
- Micro description of the phenomenon (agent’s level): simulation of the progress of infection dynamics of each patient’s disease and the transmission between them at an individual level.
- Macro description of the phenomenon: the simulation considers the population dynamics of the given microorganism.
- Spatial-temporal constraints: defined by a specific hospital building, the infection cleaning policies, and the available resources.
- Discrete event time: the simulation follows an iterative process that is divided into pre-specified periods of time (*steps)*.

**Figure 1:**
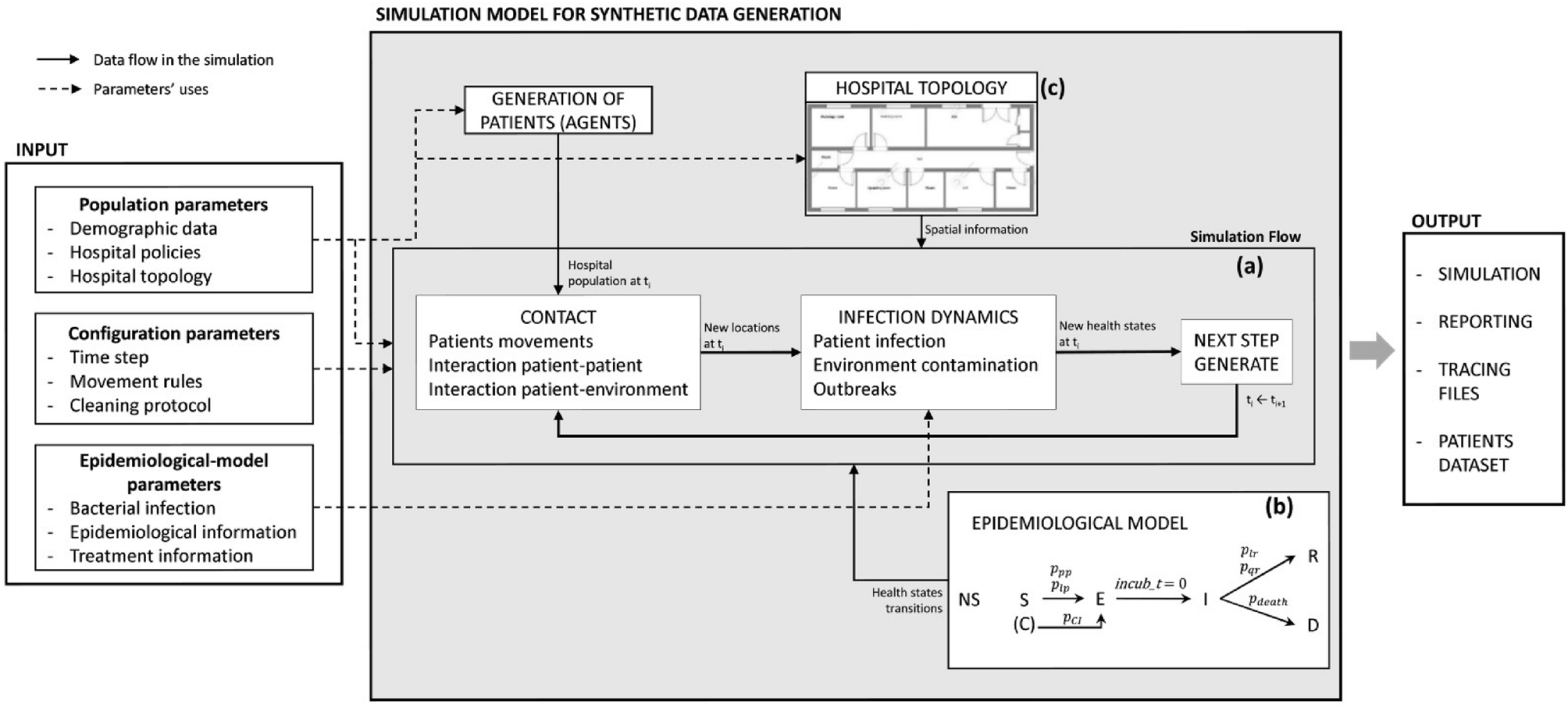
High scale representation of the simulation model with the types of input parameters and outputs. Dashed arrows represent where the input parameters are applied and solid arrows show the data flow in the simulation. The simulation (**a**) receives the patient’s health states evolution from the SEIRD epidemiological model (**b**) and the spatial constraints from the hospital topology information (**c**).

Figure 1 summarizes the high-level framework of the simulation model, describing the different types of input parameters and outputs. The elements of this framework are explained in the following subsections.

### Microscale simulation: infection dynamics at agent-level

Agent-based systems are useful for the discovery of spatial and temporal patterns thanks to the analysis at a low level. In this model, patients are the only agents: this way, we are able to study the evolution of the infection process based on solid epidemiological models, without the intervention of contagion vectors that are not verified, such as adding more agent types with different behaviors. We have considered only adult patients, that are admitted to the hospital, they move through the different areas and, eventually, they can be discharged or die.

Each patient has a unique ID to be recognized, the localization where they are, and a set of attributes: age, gender, length of stay (LOS) in the hospital, health state, incubation period, duration of infection, and applied treatment. The LOS follows a lognormal distribution and the age follows a normal distribution based on the inferences made by Codella et al.^23^.

Contact transmission is the most important and frequent mode of transmission in the healthcare setting. Organisms are transferred through direct contact between an infected (or colonized) patient and another susceptible patient (patient to patient) with probability $p_{pp}$, or by contact with the environment (patient to location). In this case, if an infected or colonized patient has spent enough time in the same place, they can contaminate it with probability $p_{pl}$. Finally, a contaminated place can infect a susceptible patient (location to patient) with probability $p_{lp}$. At the moment of any interaction, a Bernoulli experiment with said probabilities will indicate whether or not there was a contagion (more details on these probabilities in Table 1).

Regarding recovery, infected patients may have a quick recovery without treatment or a longer one with the need for treatment. Both of them have a probability of success and duration. If the patient does not recover, they may die with probability $p_{death}$.

### Macroscale simulation: compartmental model

Among the types of compartmental models^28^, the SEIRD model is the most suitable for our case since it allows us to represent a bacterial disease progression in a more exhaustive way. Thus, to represent the evolution of the disease, we have assigned each patient a state of health, that can take its value from an adaptation of the SEIRD epidemiological model. In this adaptation, we have added a new state: non-susceptible, which represents those patients that developed immunity to the disease. Patients can be in one state at a time, which could be: susceptible (S), exposed (E), infected (I), recovered (R), deceased (D), or non-susceptible (NS). Patients can arrive at the hospital in states S (i.e. normal or colonized), I or NS. The S state represents both people that can get infected, and patients who arrived colonized to the hospital (i.e. with a binary value indicating whether they are colonized or not). The latter are carriers of the bacteria and can infect others, but do not show signs of infection. An S patient can go to state E if they get infected, or if they are colonized and developed the disease, which can happen with probability $p_{CI}$. They are going to remain in state E for a period of time, meaning that they are incubating the disease, but are not contagious. For each patient, the incubation period is a random value inside a predefined range that depends on the infection. Once this period is over, the patient goes to state I, during which they can infect others and contaminate the environment. If, after all, they survive the disease, they go to state R and if not, they go to state D. Patients can be discharged from the hospital once they accomplish their LOS, and if they are in state S, NS, E, or R. This progression of the states of health can be seen in Figure 1.

### Hospital policies: spatial-temporal constraints

The space has a pertinent influence on the transmission of a disease. For this reason, within the hospital that we represented, we have focused on the areas where an inpatient is usually more likely to move and become infected. These areas are: the emergency room (ER), operating rooms, rooms for performing medical tests (e.g. X-ray, endoscope), considered as “radiology rooms” from now on, wards with several patient rooms each, and an intensive care unit (ICU). The ER, the ICU and each patient room have a user-defined number of beds. Each bed and place have a unique ID and a state indicating if they are contaminated by the infection. In case a place has been contaminated, it can expose the patients within.

The places in the hospital are divided into two types: temporary and indefinite. Temporary places are those in which a patient is going to spend a short period of time (e.g. medical tests or surgery) and, therefore, they are updated in each step of the simulation. The indefinite places are those where a patient can stay for a longer period of time (e.g. a bed, the ICU) and they are updated once per day. In order to make the movements as realistic as possible, we have constrained them spatially and temporally by means of a series of rules following the suggestions of medical experts:

- In each step of the simulation, only a limited number of patients can move to each ward.
- Patients must have spent a minimum number of steps without having gone to a temporal place to go back. For example, if a patient has just undergone surgery, they will not go back into the operating room right away.
- Patients that have been in the ER or the ICU for a certain period of time can be transferred to a ward.
- When a patient goes to a temporary place, they return to the same bed where they were before, after some fixed time defined in the simulator for each place.
- Patients can change beds in the same ward in 1 step.
- Patients in a ward can change to another ward during 1 step.
- Patients in a ward or the ER might be transferred to the ICU.

### Simulating *Clostridioides difficile* infection in a midsize hospital in Spain

The CD is currently the main cause of infectious diarrhea in hospitalized patients^29^. In recent years, cases of CD infection (CDI) within hospitals have increased, with the incidence reaching values of up to 92 per 100.000 residents in North America and Europe^29^. A person can get CDI from a carrier or through contact with a contaminated surface.

The simulations take place in a hospital environment that is based on the structure of the Rafael Méndez General University Hospital in Murcia, Spain. Our hospital has an ER with 20 beds, 3 operating rooms, 5 radiology rooms, 4 wards with 14 rooms each, 3 wards with 10 rooms each, 1 ward with 5 rooms, and an ICU with 10 beds. Each room has 2 beds and there are 212 beds in total. By comparing the size of our hospital with other real ones^30^, we have considered an area of influence of 170.000 people for this hospital.

The simulation model has a discrete time, divided into **8-hour steps**, which allows us to differentiate between 3 moments of the day: morning, afternoon, and evening. In this way, we make sure that admissions, ward changes, and discharges happen during the afternoon and only once a day. During the evening, patients can return to their beds from temporary places. Each day, a user-defined number of patients are admited through the ER or by admission to a ward in S (normal or colonized), I, or NS state. The proportion of each state was obtained from the literature^31,32^. The changes in the health state of patients and the contamination and disinfection of places can happen in each step. This disinfection of places takes place by means of a cleaning system, in which places are disinfected after a predefined number of steps. This represents how often each area is cleaned, for example, a radiology room is cleaned once a day as well as the rooms and the ICU, but beds are cleaned every 8 hours. The cleaning assiduity is based on cleaning protocol guidelines of hospitals from Spain^33^. In each step, a historic of the patients is saved with all the information about the patients and the places they are occupying at that moment (see Supplementary Tables S1and S2 online). This will be useful for the determination of epidemiological indicators and other information.

The simulation starts with an exposed (E) patient and everyone else susceptible (S). When the patients **become infected (I)**, all susceptible patients that interact with them can get infected too. Based on Sethi et al.^34^, when there are infected cases, the probability of environmental contamination via CD shedding ($p_{pl}$) is going to be higher if the infected patient has not started the treatment yet, and lower if they have been on it for more than 3 days.

Regarding the **recovery process**, there can be a quick recovery of 2-3 days after acquiring the infection, with probability $p_{qr}$ of 23%^35^. In the case of a long recovery, a treatment is applied and, during this, they can recover with probability $p_{lr}$. This treatment is configurable, both in the probabilities of success and duration. We have chosen oral Vancomycin since it has one of the highest recovery rates with a probability of 97%^36,37^, and a duration of 5 to 15 days^29^. In case of decease, a patient may die with probability $p_{death}$^38^.

### Input parameters and outputs

Input parameters can be classified into three types: population, epidemiological-model, and configuration parameters.

**Population parameters** are those that represent the population and the hospital characteristics, i.e. occupancy rate, admission rate through ER, demographic data, the hospital structure (number of beds, rooms, and wards), etc. Among these parameters, we can find the patient’s age and LOS.

**Epidemiological-model parameters** are those that depend on the bacteria that is being represented. Both these and the population parameters were obtained or calculated from public access data and information from the literature. Those parameters for which there was not enough information to infer their distribution follow a triangular distribution defined by a mean value that represents its mode, a minimum and a maximum value, based on Codella^23^.

**Configuration parameters** comprise the configuration for each run of the simulator, such as the cleaning frequency, the time that elapses before the movement of a patient depending on the place, etc. These parameters were defined based on the hospital size, data published by hospitals, and information obtained from the expert. Table 1 provides a representation of these parameters, where *max_patients_movements* encompasses the maximum number of patients’ movements allowed in the services each step, which includes Radiology, Surgery, wards, and from one room to another in the same ward; *max_time_infected* comprehends the maximum number of steps that a place is going to remain contaminated depending on the type of place and the service it belongs to; and *steps_to_infect* represents the number of steps required for an infected patient to contaminate a place, this also depends on the type of place and is provided by the expert. In Supplementary Table S3 online, these parameters are detailed with the values that we assigned for our experiments.

Regarding the **outputs**, the simulation model computes the following outputs: (1) statistics of the processes under study step by step and (2) the patients’ information. The first includes information on each patient and the places where they have been to in each step during their stay in the hospital. The second includes all the data of each patient (e.g. gender, age, admission day, etc.). More details can be found in Supplementary Tables S1 and S2 online. With this information at low level of space and time abstraction, another output is the computation of any epidemiological indicator that can be calculated with these data (e.g. prevalence, incidence density, etc.) for the different areas from the hospital.

**Table 1:** Input parameters. For triangular distributions, the mode, min. and max. parameters are presented as *mode, [min, max]*. EO = Expert Opinion.

Input type	Parameter	Value	Explanation	Source
Population	$patients\_rate$	0.7	Daily occupancy rate	^39–41^
	$arrival\_rate$	18.603	Daily arrival rate	^23^
	$p_{arrival\_ER}$	0.7	Daily arrival rate at ER	^42^
	$occupancy_{ICU}$	0.46	Occupancy rate of the ICU	^43^
	*population*	170000	Hospital area of influence	^30^
	*age*	${\overline{x}}=54, \sigma =22.52$	Patient’s age distribution	^23^
	*LOS*	4.254	Patient’s LOS mean	^23^
Epidemiological model	$arrival_S$	0.997	Prob. of arrivals in S state	^31^
	$arrival_I$	0.002	Prob. of arrivals in I state	^31^
	$arrival_{NS}$	0.001	Prob. of arrivals in NS state	^31^
	$arrival_{C}$	0.076	Prob. of arrival in colonized state over the whole population	^23^
	$p_{pl}$	0.52, [0.14, 0.9]	Prob. of patient infecting place. Triangular distribution	^34^
	$p_{lp}$	0.435, [0.326, 0.544]	Prob. of place infecting patient. Triangular distribution	^23^
	$p_{pp}$	0.24, [0.18, 0.3]	Prob. of patient infecting patient. Triangular distribution	^23^
	$p_{CI}$	0.0114, [0, 0.0227]	Prob. of colonized patient becoming infected. Triangular distribution	^23^
	$incubation\_time$	[48, 72]	Min. and max. incubation period (hours)	^44^
	$p_{qr}$	0.115, [0, 0.23]	Prob. of quick recovery. Triangular distribution	^35^
	$p_{lr}$	0.798, [0.599, 0.998]	Prob. of long recovery. Triangular distribution	^23^
	$treatment\_days$	10, [5, 15]	Treatment duration. Triangular distribution	^23^
	$p_{death}$	0.027	Prob. of death	^45^
Simulation configuration	$step\_time$	8	Step duration (hours)	Normal workday
	$max\_patients\_movements$	Depends on hospital beds	Max. number of patients allowed by service per step	EO
	$max\_time\_infected$	Depends on hospital beds	Max. infection duration of each place	EO^33^
	$steps\_to\_infect$	Depends on place	Number of steps required to infect a place	EO

### Evaluation

For the evaluation process, we have followed the principles proposed by Banks et al.^46^. In their handbook, they divide the evaluation process into 2 phases: verification and validation. Verification consists in checking if the implementation of the conceptual model is correct. Validation consists in determining if the conceptual model is representative of a real-world system.

We implemented the simulator in Python, using the PyCharm IDE 2022.3.1 version. By means of this environment, we carried out the following **verification** techniques:

- **Syntax analysis:** to ensure that the mechanics of the language were applied correctly.
- **Debugging:** through which we have checked errors in the coding that caused the simulator to fail.
- **Execution testing:** we analyzed the simulator behavior to find errors in the model representation. We traced the movements of the agents around the hospital, as well as the changes in their health states and the infection state of the places. To do this, we have implemented a 3D representation of the hospital in the game engine Unity and we have analyzed the patients’ behavior throughout their stay by means of **animations**. An example of these animations can be found in Supplementary File S3

With respect to **validation**, the main technique for the validation of a simulation model is the comparison with real data. As we have already mentioned, one of the big issues we face is the lack of access to them. Therefore, our validation process consisted of the following phases:

- **Face validation:** a domain expert confirmed whether the model was behaving reasonably, judged whether it was accurate enough^46^, and advised us on the input parameters and the model assumptions that we have made.
- **Calibration of input parameters:** to test values for a parameter iteratively until the model is valid enough^46^. We have adjusted the population and the epidemiological-model parameters regarding the data available in the literature, as well as the configuration parameters with the help of an expert, with the aim of getting a simulation model with behavior as close as possible to that of an already validated one.
- **Model alignment with Bootstrapping:** through which we compared the model results with those of another previously validated one using the same input data^46,47^. If the results of both models are similar, then the generated model (i.e. our simulation model) produces the general dynamics that would be expected in the system to be modeled^48^. In the simulation of infectious diseases, a standardized method for their representation are compartmental models, where the population dynamics are well known^48^. To this end, we have compared the results derived from our model with the SEIRD (Susceptible, Exposed, Infected, Recovered, Deceased) model^28^, which is a deterministic compartmental model that we had to adapt in order to represent the same behaviour as the population from our simulation model. Thus, we have added the non-susceptible (NS) state, obtaining the SEIRD-NS model, and we had to make the population dynamic: in the classic SEIRD model, the total population N is a constant value, i.e. $N = S+E+I+R+D$. In our case, as we worked with a hospital where there are admissions and discharges, the population is going to change. This model is presented in Eq. (1). The arrival rate per day (*a*), the proportions of arrivals in each state ($a_s, a_i, a_{ns}$) and the mortality rate ($\mu$) are explained in Table 1, while the discharge rate (*d*) is defined as the inverse of the length of stay (LOS)^16^. The recovery rate ($\gamma$) is defined as $1 - \mu$, the average incubation period (Table 1) is defined as $\alpha ^{-1}$, and the contact rate ($\beta$) is defined as the inverse of the probability of getting infected (Table 1).
  To compare the output data of the SEIRD-NS model with the outputs of our simulator we have applied statistical techniques as indicated by^46^. To do so, we have calculated the confidence intervals with as few runs of the simulator as possible in order to get the value range of each health state of the patients and to compare them with the SEIRD-NS model. In this way, we are going to see if the simulator output has a trend close enough to the SEIRD-NS model to be considered valid.
  To calculate the confidence intervals, we ran the model a high number of times (1000 executions) and made incremental groupings of runs (10, 20, 30, ..., 1000). For each group, we recorded the number of patients in each health state at different moments of the execution (on days 20, 40, 60, etc.). And for each of those moments and grouping of runs, we determined the confidence interval for the mean and compared it with the objective model. The equation of the confidence interval for the mean is $CI = {\overline{x}} \pm z * \frac{s}{\sqrt{n}}$, where ${\overline{x}}$ is the mean of the sample we want to compare, *z* is 1.96 for a 95% confidence interval, *s* is the standard deviation of the sample and *n* is the sample size (in our case, the number of runs). In this way, we compared the simulator output for the number of people in S, E, I, R, D, and NS states for each group of executions and each time partition with the SEIRD-NS model for the same partition of time.
- **Sensitivity analysis:**we observed how changes in certain input parameters affected the model and its output, and so inferred if the outcomes were realistic or not. To this end, we have obtained the incidence density (DI) from the output. The equation of the incidence density is $DI = newCases / totalPerson-time$, where *totalPerson-time* is the sum of the total time at risk among all the patients during the observation period.

1 $$\begin{aligned} \begin{aligned}{}&dS/dt = -\beta *S*I/N + a_s*a - d*S&dE/dt = \beta *S*I/N - \alpha *E - d*E\\&dI/dt = \alpha *E - \gamma *I - \mu *I + a_i*a&dR/dt = \gamma *I - d*R \\&dD/dt = \mu *I - D&dNS/dt = a_{ns}*a - d * NS \\ \end{aligned} \end{aligned}$$

## Results

**Figure 2:**
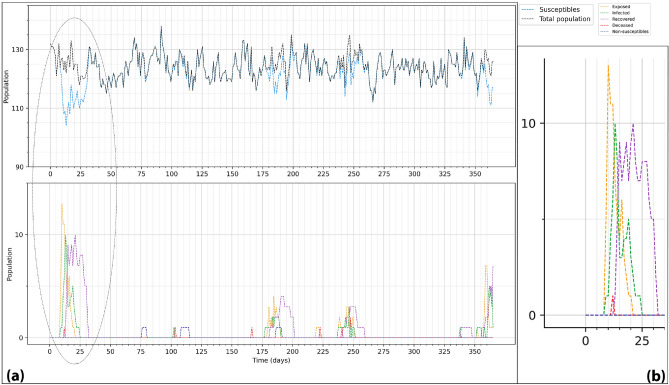
(**a**) Macro-scale model output with several outbreaks of CDI. This figure shows the infection dynamics and number of patients in each health state. The first outbreak is highlighted. (**b**) Detailed description of the evolution of an outbreak.

In this section, we present the results of the calibration, the model alignment, and the sensitivity analysis processes. In Figure 2 we can see part of the output from a run of our simulator with CDI, which represents the number of patients in each health state and the dynamics and evolution over time. In this Figure, we highlight the first outbreak to better demonstrate the evolution of the states.

### Calibration of parameters

**Figure 3:**
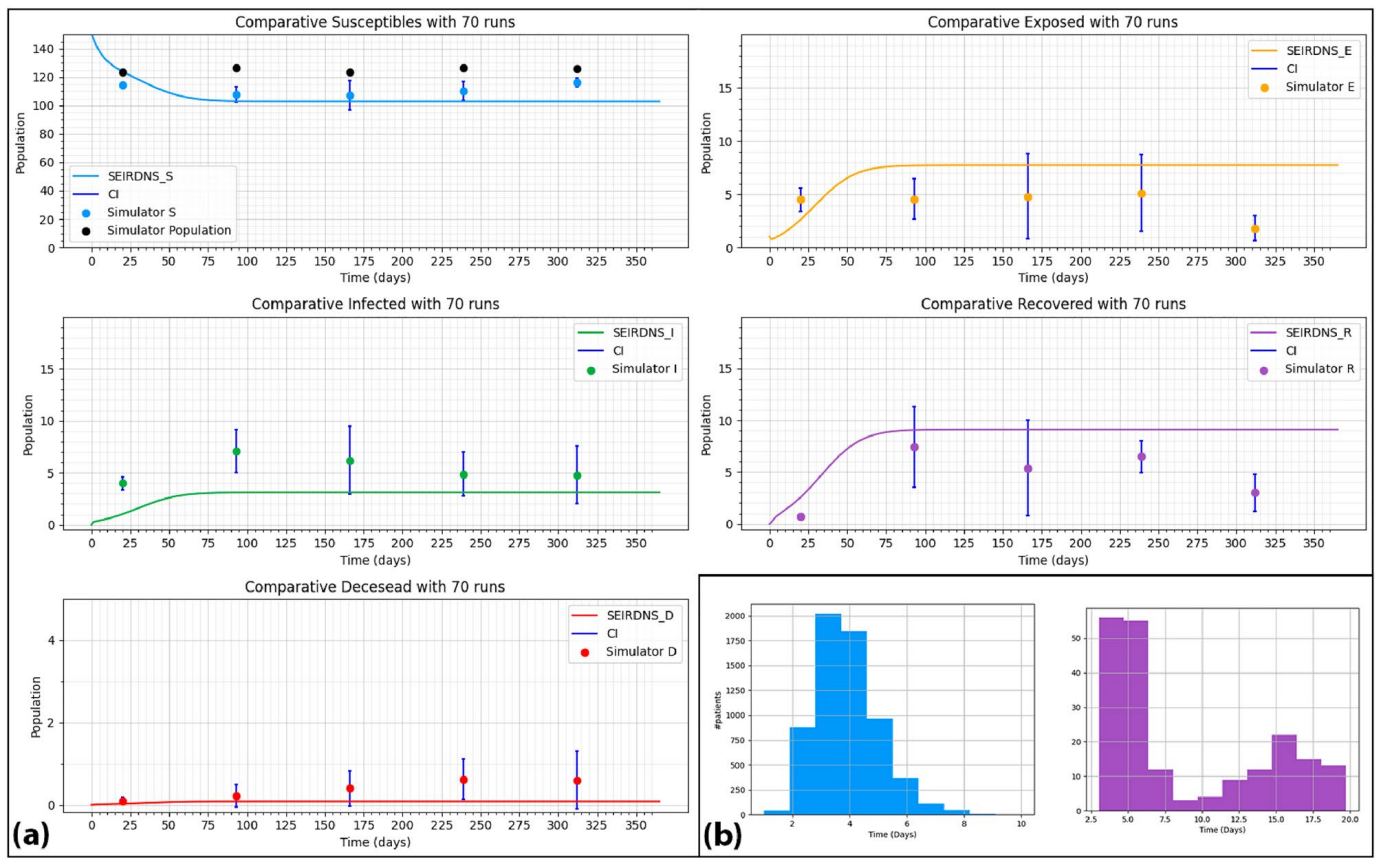
(**a**) Model alignment of the simulation model outputs (scatter plot) with the SEIRD-NS compartmental model (line chart). The scatters show the mean of the outputs with 70 runs for each state of health and each point of time with their confidence intervals (CI). The SEIRD-NS compartmental model is obtained with Eq. 1. (**b**) Comparison of Length of stay of susceptible patients (blue) vs length of stay of recovered patients (purple) for the validation of the calibration parameter process.

We have based on validated scientific literature for the calibration of the input parameters, and we have conducted an iterative process of validation with an expert. The results from this process are presented in Table 1. To verify the adjustment of these parameters, we have compared the outputs of the simulator with published literature on CDI. In a study carried out by Barbut and Petit^49^ on the epidemiology of infections associated with CD, they indicated that the LOS of patients infected with CD is increased between 8 and 21 days. In Figure 3 (bottom right), we show the LOS of susceptible and recovered patients from our simulations: for those patients that recovered from the infection, their LOS has properly increased, reaching values between 8 and 22 days. The proximity of the LOS results to the values reported by Barbut indicates that the infection behavior in the patients in our simulation model is valid.

### Model alignment

As the SEIRD-NS compartmental model represents the dynamics of a population when an outbreak occurs and the successive contagions until the situation stabilizes again, we have considered only the runs from the simulator where at least an outbreak has happened since due to the stochasticity of agent-based models there can be executions without contagions. Thus, we have based on the definition of a CDI outbreak by^50^, where they define a CDI outbreak as 3 or more epidemiologically linked cases that appear in a period of 7 days or less.

When comparing the output of our model with the SEIRD-NS model, another issue was the moment when we should start the comparison: if, for example, the outbreak started on day 100 of the simulation, it would not make sense to compare from day 1 to 100 with a SEIRD-NS model whose first outbreak was on day 10. Therefore, we had to align the beginning of the outbreak in the compartmental model with the beginning of the outbreak in the simulator outputs. To do this, we considered the first day of the run to be the first day on which an outbreak appeared, and from there we began to compare. Both models were initialized with a total population of susceptibles and 1 patient exposed. The results of this comparison are shown in Figure 3. We can see that for 70 runs, the sizes stay relatively the same and small enough in the number of individuals. This allows us to analyze the trends of the simulator runs with outbreaks and, though both models do not return the same number of patients in each state on each day, the infection dynamics are similar.

### Sensitivity analysis

For the sensitivity analysis, we chose to alter the cleaning protocol and the time that patients spend in more crowded places to create the following scenarios: a) a scenario without cleaning service – the infected places will remain contaminated until the end of the simulation–; b) a scenario in which we increased the cleaning frequency so that each room, bed and ward is decontaminated once every step and the ER once a day; c) a scenario in which the patients spend more steps in the ER or the ICU; and d) a scenario in which the patients spend less steps in the ER or the ICU. As a comparative measure, we have obtained the DI from our simulator, which returned a value of 18.92 (16.96, 20.89) *cases/10000 patient-days* in the original scenario. As expected, in the scenario with no cleaning service, the DI increased significantly to a value of 1327.89 (1268.72, 1387.06) *cases/10000 patient-days*, while when we increased the cleaning frequency, the DI decreased compared to the original situation to a value of 15.78 (13.81, 17.75) *cases/10000 patient-days*. This agrees with^51,52^ about the need of sterilizing the environments to control CD outbreaks. With respect to the time spent in more crowded places, in the scenario with more time, the DI increased to 53.11 (44.48, 61.75) *cases/10000 patient-days*. While by spending less time, the DI was slightly decreased to 17.35 (15.44, 19.27) *cases/10000 patient-days*. As patients stay more time in places with more beds, the spread of the infection increases correctly.

## Discussion

The need to access and use clinical data without risk or limitations is a long-term issue that has slowed down the development of AI innovations that could help in a large number of tasks in the healthcare field. Due to this problem, we have developed a simulator to generate synthetic data of inpatients in hospitals. The aim of this simulator is to allow the study of the spread and outbreaks of microorganisms of epidemiological relevance and other MDR-bacteria as CD infections within hospital settings. To this end, we have created a simulation model with which we could represent the dynamics between patients and the environment, and we gave special weight to the role that plays the topology of a hospital in the spread process. For this reason, we have highlighted the main areas and wards where there are usually contagions and outbreaks according to experts in the field, as the space must be a key element in the epidemiological investigation of an outbreak.

An advantage of using our agent-based model over the use of real data is the generation of information with a higher level of detail. This allows us to trace the patients spatially and temporally: thanks to the historic data of patients we can know their entire spatial, temporal and clinical chronology (i.e, where they have been at any moment and what infections or health states they have experienced since their arrival to the hospital). Therefore, we can simulate specific situations and obtain accurate results, for example, the DI, for which it is necessary to know the days that a patient spends without getting infected after admission. The latter cannot be monitored with that level of detail in a real situation normally, this is why the DI is usually determined considering the total stay of a patient or the total observation time. This is also the reason why we cannot compare our results with the literature, since, at least by the time of submitting this article, we could not find in the literature CDI outbreaks with continuous monitoring as in our model, and so the results would differ.

Besides accurate monitoring, another advantage of this simulation model is that it allows for predicting information and statistical data about the infection, namely, the exact duration of an infection depending on the treatment, how much this is going to increase the patient’s LOS, or the patients’ time-at-risk since they are hospitalized. Thanks to this simulation model, we can predict this type of data and perform sensitivity analysis that would help to measure the effects of control strategies and to plan the allocation of limited resources, such as isolated beds in a hospital and the types of isolation. For example, in the sensitivity analysis we studied several scenarios by varying the time patients spend in crowded places and the cleaning assiduity, which are fundamental factors in an infection spread. From this, we could infer that without isolation, a longer contact time with the source of infection has more weight in the process of contamination than the movement of patients or more occasional contacts. Other factors that affect patients within a hospital could be introduced and studied with this simulation model, such as viral infections.

Several aspects differentiate our simulation model from existing HAIs processes simulators in the literature. Codella et al.^23^ developed an agent-based with a Markov model to study the transmission of CD in a mid-size hospital. Unlike them, the principal goal of our simulation model is to generate realistic spatial-temporal datasets with individual information for their latter use in AI implementations. Lee et al.^25^ presented a software tool, where practically any healthcare facility type can be represented to help test different policies. Instead of this, we focus more on the role of space on an infection spread by studying the latter in the most common areas of a hospital environment (e.g. the ER, the ICU, etc.). Another difference is that they use subroutines to calculate the number of infected and susceptible agents in each ward, and based on that, they calculate the number of new cases by ward and day. Instead of this, we monitor all the agents present in the hospital, so that we can know when they shared a room and interacted at low level. Haber et al.^26^ combined an agent-based with a compartmental model and focused on the study of second-line drugs. They calculate the infection spread with differential equations and they do not consider patients movements in the hospital, nor give the same importance to space and time as we do. Despite Nelson et al.^24^ also carried out a model with interactions between patients and healthcare workers, room contamination, and patient infection, they focused on conducting an economic evaluation of different scenarios.

This work is not without limitations. As we mentioned, due to the lack of access to real data, some of our parameters are approximations made from a few data sources. Despite the fact that CDI is a well-studied and well-known infection, the literature is scarce for the level of information that we need for this model. Moreover, we did not consider relapses of the patients, in order to be able to adapt it as much as possible to the health states and transitions of a SEIRD model for the evaluation. For this reason, we have used an average mortality rate rather than differentiating by ranges of age, as happens in reality and as was indicated by Loo et al.^38^. Since there are few specific studies in the literature, we have relied in part on^23^ and expanded it with respect to the role that space plays in the simulation and the movement rules.

This simulation model is implemented to endure high-scale data, different population sizes and hospitals. It serves as a starting point to create other more complex models that would allow richer analysis. For example the creation of input risk profiles of patients according to age or previous treatments; the implementation of different treatments that could vary in their success rate and duration, and allow for choosing one or another regarding the case; or the study of other CD strains or other MDR bacterial infections and the consequences of applying different strategies to mitigate them. The application of data with this level of detail in AI research has potential benefits, such as the reduction of subjectivity in decision-making and for helping to control risk situations.

## Conclusions

We have designed a simulation model and implemented a simulator based on this, that coupled an agent-based approach with the infection dynamics extracted from an epidemiological compartmental model, with the aim of creating a generator of synthetic clinical data on microorganisms of epidemiological relevance as CD and other MDR-bacteria within hospitals. Thanks to the use of an agent-based method and the role that plays the hospital topology in the simulation model, we can leverage the detection of spatial and temporal patterns to help in the monitoring and in the decision-making process. We have carried out a thorough evaluation to ensure that it was correctly implemented, and had clinical meaning and utility. The capacity of tracing patients at a low level and to also obtain aggregate results from them can play a key role and be a step forward in the generation of higher-quality synthetic data and the creation of a more trustworthy medical AI.

### Supplementary Information

Supplementary Information.

## Data Availability

The simulation model source code and datasets generated are available in the Simulation Model repository at https://github.com/denissekim/Simulation-Model.
