# Supplementary material for: Spatial-temporal simulation for hospital infection spread and outbreaks of Clostridioides difficile
Source: Sci Rep. 2023 Nov 16;13:20022. doi: 10.1038/s41598-023-47296-1 (PMC10654661; doi:10.1038/s41598-023-47296-1)
Supplement: Supplementary file 1 — Supplementary Information. [file 41598_2023_47296_MOESM1_ESM.zip › Supplementary files/File S4.docx]

Figure 1: Verification of simulation model. Verification of the correct functioning of the simulation model through animations in the Unity framework.
