# Supplementary material for: Spatial-temporal simulation for hospital infection spread and outbreaks of Clostridioides difficile
Source: Sci Rep. 2023 Nov 16;13:20022. doi: 10.1038/s41598-023-47296-1 (PMC10654661; doi:10.1038/s41598-023-47296-1)
Supplement: Supplementary file 1 — Supplementary Information. [file 41598_2023_47296_MOESM1_ESM.zip › Supplementary files/Supplementary Tables S1 and S2.pdf]

Table S1. Example of information of the patients that stayed in the hospital. Colonized and NS indicates whether the patient was admitted in those conditions. NS = Non Susceptible, LOS = Length of Stay, T = Duration, Inc = incubation, Tx = Treatment, Adm = Admission

ID	Age	Sex	Colonized	NS	LOS	T Inc.	T Infection	T Tx	Adm. day	Last day
0	45	M	False	False	5 days	54 hs	0	0	0	3
1	62	M	False	False	4 days	70 hs	0	0	0	6
2	41	F	False	False	3 days	72 hs	80 hs	8 days	1	7

Table S2. Example of trace of the patients' health state and location in the hospital. Patient State can have the following values: 0 (S), 1 (E), 2 (I), 3 (R), 4 (D), 5 (NS).

Step	Patient ID	Patient State	Location ID	Location Infected
0	0	1	216	False
0	1	0	189	False
0	2	0	29	False
