# Supplementary material for: Spatial-temporal simulation for hospital infection spread and outbreaks of Clostridioides difficile
Source: Sci Rep. 2023 Nov 16;13:20022. doi: 10.1038/s41598-023-47296-1 (PMC10654661; doi:10.1038/s41598-023-47296-1)
Supplement: Supplementary file 1 — Supplementary Information. [file 41598_2023_47296_MOESM1_ESM.zip › Supplementary files/Table S3.pdf]

Table S3. input parameters itemized. For triangular distributions, the mode, min. and max. parameters are presented as *mode, [min, max]*. ER = Emergency Room, ICU = Intensive Care Unit, LOS = Length of Stay, OR = Operating Room.

Input type	Parameter	Value	Explanation	Source
Population	$patients\_rate$	0.7	Daily occupancy rate	[39–41]
	$arrival\_rate$	18.603	Daily arrival rate	[23]
	$p_{arrival\_ER}$	0.7	Daily arrival rate at ER	[42]
	$occupancy_{ICU}$	0.46	Occupancy rate of the ICU	[43]
	$population$	170000	Hospital area of influence	[30]
	age	$\bar{x} = 54, \sigma = 22.52$	Patient's age distribution	[23]
	LOS	4.254	Patient's LOS mean	[23]
Epidemiological model	$arrival_S$	0.997	Prob. of arrivals in S state	[31]
	$arrival_I$	0.002	Prob. of arrivals in I state	[31]
	$arrival_{NS}$	0.001	Prob. of arrivals in NS state	[31]
	$arrival_C$	0.076	Prob. of arrival in colonized state over the whole population	[23]
	p_{pl}	0.52, [0.14, 0.9]	Prob. of patient infecting place. Triangular distribution	[34]
	p_{lp}	0.435, [0.326, 0.544]	Prob. of place infecting patient. Triangular distribution	[23]
	p_{pp}	0.24, [0.18, 0.3]	Prob. of patient infecting patient. Triangular distribution	[23]
	p_{CI}	0.0114, [0, 0.0227]	Prob. of colonized patient becoming infected. Triangular distribution	[23]
	$incubation\_time$	[48, 72]	Min. and max. incubation period (hours)	[44]
	p_{qr}	0.115, [0, 0.23]	Prob. of quick recovery. Triangular distribution	[35]
	p_{lr}	0.798, [0.599, 0.998]	Prob. of long recovery. Triangular distribution	[23]
	$treatment\_days$	10, [5, 15]	Treatment duration. Triangular distribution	[23]
	p_{death}	0.027	Prob. of death	[45]
Simulation configuration	$step\_time$	8	Step duration (hours)	Normal workday
	$steps$	1095	Duration of simulation (1 year)	
	$max\_patients_{rx}$	20	No. patients allowed in Radiology per day	EO
	$max\_patients_{sx}$	15	No. patients allowed in OR per day	EO
	max_moves_{wards}	2	Max No. of allowed movements inside a ward per day	EO
	max_moves_{room}	5	Max No. patients that can change to a room per day	EO
	$max\_steps_{ER\_ICU}$	3	Steps that a patient has to remain in ER or UCI, for being changed to a room	EO
	min_steps_{rx}	10	Min steps that a patient has gone through without going to Radiology to be able to return	EO
	min_steps_{sx}	10	Min steps that a patient has gone through without going to OR to be able to return	EO
	$max\_time\_infected_{rx}$	3	Steps that a radiology room will remain infected	EO,[33]

Continued on next page

Table S3 – continued from previous page

Input type	Parameter	Value	Explanation	Source
	$max\_time\_infected_{bed}$	1	Steps that a bed will remain infected	EO,[33]
	$max\_time\_infected_{room}$	3	Steps that a ward room will remain infected	EO,[33]
	$max\_time\_infected_{ICU}$	3	Steps that the ICU will remain infected	EO,[33]
	$max\_time\_infected_{ward}$	3	Steps that a ward will remain infected	EO,[33]
	$max\_time\_infected_{ER}$	6	Steps that the ER will remain infected	EO,[33]
	$max\_time\_infected_{OR}$	1	Steps that an OR will remain infected	EO,[33]
	$steps\_infect_{rx}$	1	Steps that a patient has to be in a radiology room to be able to infect it	EO
	$steps\_infect_{sx}$	1	Steps that a patient has to be in an OR to be able to infect it	EO
	$steps\_infect_{bed}$	1	Steps that a patient has to be in a bed to be able to infect it	EO
	$steps\_infect_{room}$	2	Steps that a patient has to be in a ward room to be able to infect it	EO
	$steps\_infect_{ICU}$	2	Steps that a patient has to be in the ICU to be able to infect it	EO
	$steps\_infect_{ward}$	2	Steps that a patient has to be in a ward to be able to infect it	EO
	$steps\_infect_{ER}$	1	Steps that a patient has to be in the ER to be able to infect it	EO
	$steps\_infect\_p_{rx}$	1	Steps that a patient has to be in a radiology room to be infected by it	EO
	$steps\_infect\_p_{sx}$	0	Steps that a patient has to be in an OR to be infected by it	EO
	$steps\_infect\_p_{bed}$	0	Steps that a patient has to be in a bed to be infected by it	EO
	$steps\_infect\_p_{room}$	1	Steps that a patient has to be in a room to be infected by it	EO
	$steps\_infect\_p_{ICU}$	1	Steps that a patient has to be in the ICU to be infected by it	EO
	$steps\_infect\_p_{ward}$	1	Steps that a patient has to be in a ward to be infected by it	EO
	$steps\_infect\_p_{ER}$	1	Steps that a patient has to be in the ER to be infected by it	EO
